# Electroactive *Brevundimonas diminuta* consortium mediated selenite bioreduction, biogenesis of selenium nanoparticles and bio-electricity generation

**DOI:** 10.1186/s12951-024-02577-3

**Published:** 2024-06-20

**Authors:** Ebtehag A. E. Sakr, Dena Z. Khater, Kamel M. El‑khatib

**Affiliations:** 1 Botany Department, Faculty of Women for Arts, Science and Education, Ain Shams University, Cairo, Egypt; 2 Chemical Engineering and Pilot Plant Department, Engineering Research and Renewable Energy Institute, National Research Centre (NRC), El Buhouth St, Dokki, Cairo, 12622 Egypt

**Keywords:** *Brevundimonas diminuta*, Selenite-reducing bacteria, Selenium nanoparticles, Bioremediation, Microbial fuel cell, Electrochemical activity

## Abstract

In this study, highly selenite-resistant strains belonging to *Brevundimonas diminuta* (OK287021, OK287022) genus were isolated from previously operated single chamber microbial fuel cell (SCMFC). The central composite design showed that the *B. diminuta* consortium could reduce selenite. Under optimum conditions, 15.38 Log CFU mL^-1^ microbial growth, 99.08% Se(IV) reduction, and 89.94% chemical oxygen demand (COD) removal were observed. Moreover, the UV–visible spectroscopy (UV) and Fourier transform infrared spectroscopy (FTIR) analyses confirmed the synthesis of elemental selenium nanoparticles (SeNPs). In addition, transmission electron microscopy (TEM) and scanning electron microscope (SEM) revealed the formation of SeNPs nano-spheres. Besides, the bioelectrochemical performance of *B. diminuta* in the SCMFC illustrated that the maximum power density was higher in the case of selenite SCMFCs than those of the sterile control SCMFCs. Additionally, the bioelectrochemical impedance spectroscopy and cyclic voltammetry characterization illustrated the production of definite extracellular redox mediators that might be involved in the electron transfer progression during the reduction of selenite. In conclusion, *B. diminuta* whose electrochemical activity has never previously been reported could be a suitable and robust biocatalyst for selenite bioreduction along with wastewater treatment, bioelectricity generation, and economical synthesis of SeNPs in MFCs.

**Supplementary Information:**

The online version contains supplementary material available at 10.1186/s12951-024-02577-3.

## Introduction

Chalcogenic selenium is considered a rare earth elements of the earth’s crust that are mostly found in the environment as hazardous oxyanions. It is a necessary micronutrient for biological systems at lower doses but turns poisonous at higher amounts [1]. Selenium (Se) has extensive applications in industrial fields like solar cells, rubber vulcanization, and electrolysis [2]. Also, it contributes to the health of both people and animals because of its immunomodulatory, anti-oxidant, and anti-cancer properties [2–4]. The World Health Organization (WHO) suggests the perfect consuming dose is from 40 to 400 µg of selenium per day. However, the US Environmental Protection Agency (UEPA) sets the maximum amount of Se that can be contaminate wastewater at less than 50 µg of Se per liter in drinking water [5, 6]. Globally, Se pollution is linked to numerous human endeavors such as refining oil, burning coal, fossil fuels, weathering and leaching rocks, extracting and processing crude oil, mining, landfills, and agriculture [7, 8]. There are four main valence states for selenium: elemental selenium (Se^0^), selenite (SeO_3_^2−^), selenide (Se^2−^), and selenate (SeO_4_^2−^) [9]. Se oxyanions (SeO_3_^2−^ and SeO_4_^2−^) are considered as highly toxic soluble forms of Se that can have harmful impacts on human health that leads to heart, skin, and neurological problems [1, 10]. Conversely, Se^0^ is thought to be somewhat harmless because of its low solubility and low toxicity [11].

The removal of Se from industrial wastewater by physicochemical techniques is challenging and expensive [12]. Therefore, innovative and unique remediation technologies are required in order to provide a safe, affordable, and environmentally friendly bioremediation of contaminated Se sites. Among these approaches are microbial fuel cells (MFCs) that are bioelectrochemical systems (BES) or bioelectrochemical treatment systems (BET), they can be used for the removal and recovery of metal ions from metallurgical wastes, process streams and wastewaters [13–15]. They have been effectively combined with cathodic reduction of metal ions to generate electricity by the biodegradation of organic matter by electroactive anodic biofilms [16–18]. Transforming soluble and hazardous oxyanions into insoluble Se^0^ is a bioremediation strategy that aims to clean wastewater and recover resources simultaneously [19–22]. The microbe-electrode and metal interactions that serve as terminal electron acceptors (TEAs) in the MFCs are essential for recovering rare metals and preserving regulatory levels [6, 23]. Few studies demonstrated how bacteria can undergo both aerobic and anaerobic conditions to characterize the selenite bioreduction that is less toxic via anaerobic respiration and detoxification [24–26]. Catal et al. (2009) assessed the efficacy of single-chamber MFCs (SCMFCs) using glucose or acetate as an electron donor. The study demonstrated that the coulombic efficiencies of SCMFCs rose from 25 to 38% at 150 mg L^− 1^ of Se [22].

Some microorganisms are able to bioaccumulate, biomethylate, or bioconvert selenium ions [27]. The bacterial pure cultures, bacterial biofilms, and microbial consortia grown in reactors with various topologies are used for the biological reduction of selenium oxyanions from wastewater, soils, and sediments [10].

Selenium can be reduced into elemental selenium nanoparticles (SeNPs) biologically when a carbon source available to act as an electron donor and the bioconversion can occur in an anoxic environment [28, 29]. Afterward, the resulting SeNPs might be recovered as a valuable resource to be used in semiconductors, glass manufacturing, biomedicine, solar cells, alloys, light-emitting diodes, and biological imaging, among other uses [1, 10, 27]. Microorganisms are therefore appealing as potential nanofactories for the environmentally friendly biogenic synthesis of SeNPs. As a result, there is an immediate need for novel strains with high Se tolerance or quick Se metabolism.

To our knowledge, this was the first report on simultaneous reduction of selenite and COD by *B. diminuta* in a SCMFC. The SCMFC had the scope and potential to emerge as a green route for the simultaneous removal and recovery of metals along with wastewater treatment and bioelectricity generation. The objectives of this study were demonstrated as follow, firstly, isolation and identification of selenite-reducing bacteria from previously operated SCMFC. Secondly, optimizing the effective factors via response surface method (RSM) on the growth biomass, bioremediation process of selenite, and organic matter. Thirdly, characterization of resulted SeNPs using UV, FTIR, and TEM. Finally, the bioelectrochemical performance of SCMFC towards biogenesis of SeNPs was also evaluated.

## Materials and methods

### Isolation of selenite reducing electroactive bacteria

After SCMFC had been operating for three months, the bacteria were isolated from the anodic and cathodic biofilm. The original inoculant for the previous study was activated sludge from a Zenien wastewater treatment plant (Bolaq Aldakrur, Giza, Egypt) [30]. Separate inoculations of both electrodes (1.0 × 1.0 cm) were made in 100 mL of nutrient broth media which were then allowed to adjust to pH 7.5 at 37 ^o^C for 48 h. After being serially diluted, the bacterial suspensions were spread out on nutrient agar (NA) that contained 300 mg L^-1^ from Na_2_SeO_3_. To obtain pure bacterial monocultures, the individual colonies were then streaked on fresh media. In order to examine these isolates’ capacity to reduce selenite to red elemental Se^0^, their growth tolerance was assessed on NA media plates supplemented with varying concentrations of Na_2_SeO_3_ (100–700 mg L^–1^). After the color of the culture changed to red, it was an indicator of the selenite reduction into SeNPs. The selected colonies were kept for additional trials at 4 °C on NA medium plates enriched with Na_2_SeO_3_.

### 16s rRNA gene sequencing and phylogenetic analysis

DNA extraction was done at Sigma Company using the pure colonies of the isolates that could tolerate the highest levels of stress on agar plates. Using the polymerase chain (PCR) reaction, the 16 S ribosomal RNA gene was amplified using the universal 27 forward primers (5’AGA GTT TGA TCC TGGCTC AG3’) and 1492 reverse primers (5’GGY TAC CTT GTTACG ACT T3’). Following PCR, the results were purified and sequenced. The retrieved sequences were subjected to BLAST search and compared with closely related species’ sequences that obtained from the GenBank database. The maximum likelihood technique was then used in MEGA 11 to develop a phylogenetic tree. The sequences were submitted to the GenBank database.

(https://www.ncbi.nlm.nih.gov/nucleotide/OK287021.1?report=genbank&log$=nuclalign&blast_rank=1&RID=TWVDBTV1016;https://www.ncbi.nlm.nih.gov/nucleotide/OK287022.1?report=genbank&log$=nuclalign&blast_rank=1&RID=TWVNJWGH01N).

### Statistical optimization

Response surface method (RSM) contributed in optimizing the growth biomass, chemical oxygen demand (COD) removal efficiency and selenium removal in analyzing the relationship between these parameters. The responses could be attributed to four independent variables: (A) selenite concentration, (B) sodium acetate (electron donor), (C) inoculum size of anodic bacterial isolate and (D) inoculum size of cathodic bacterial isolate. To assess the statistical validity of the fitted model and the model’s lack of fit, there are sufficient experimental points in the central composite design (CCD). All variables were examined at five levels in full factorial CCD (-α, -1, 0, + 1, +α). Using four components at five levels of independent variables and six repeating central points, thirty run formulations were produced (Table 1). The experiments were set up in accordance with the design and carried out in 250 mL Erlenmeyer flasks containing 100 mL of media for 48 h at 37 ^o^C without agitation. The equations that were generated provide information on the importance of the aforementioned parameters. The design was created and examined using the statistical software “Design-Expert® 10” (Stat-Ease Inc., Minneapolis, USA) program. Multiple regression analysis was used to create response surface graphs, which show how every significant factor interacts with the others to evaluate the optimal medium components. The effect and regression coefficients of the individual linear, quadratic, and interaction factors were determined using statistical analysis (ANOVA). Fisher’s test was used to determine the model equations and the model terms’ statistical significance. The coefficient of determination (R^2^) was utilized to express the degree of quality of fit for the second-order polynomial model [31].

In order to estimate biomass, the culture was inoculated into NA plates using the pour method, serially diluted with sterile saline, and then incubated at 37 ^o^C for 48 h. The number of growing cells was counted (Log CFU mL^− 1^). In order to analyze chemical oxygen demand (COD), samples were centrifuged for 20 min at 6000 rpm. The supernatant was then used to calculate COD using the closed reflux colorimetric method, which is a standard process recommended by the American Public Health Association.

For analysis of selenite concentration [32], after the liquid samples were collected at the ending of the experiment, they were centrifuged for 20 min at 6000 rpm to exclude the bacterial cells and Se^0^. The supernatant was then combined with 1 mL of 1 M ascorbic acid and 0.5 mL of 4 M HCl. After 10 min of incubation at room temperature, absorbance of the mixture was evaluated at 500 nm using a UV–Vis spectrophotometer. Selenium removal efficiency (R) was estimated as follows: Removal efficiency (%); R = [(C_0_ − C_t_)/C_0_] * 100, where, C_0_ = Initial concentration (mg L^− 1^) and C_t_ = Concentration at time t (mg L^− 1^) [33].

### Characterization of SeNPs

For characterization of SeNPs produced due to selenite bio-reduction, the bacterial cells were cultured in an optimal nutrient broth fortified with 350 mg L^− 1^ Na_2_SeO_3_ for 48 h at 37 °C. The bacterial cultures were incubated, and then the pellets were collected by centrifuging them for 10 min at 6,000 rpm. After two rounds of 0.9% NaCl rinsing, the pellets were again suspended in 20 mL of Tris-HCl buffer (50 mM, pH 8.2). SeNPs were then extracted from the samples after they endured ultra-sonication treatment [34]. A spectrophotometer (Shimadzu UV-1800) was used to record the UV–visible absorption spectra of the SeNPs, covering a wavelength range of 200–600 nm. Using an FT-IR spectrophotometer (Bruker Alpha 11), the functional groups of SeNPs were determined. In absorbance mode, mid-infrared spectroscopy (4000–400 cm^− 1^) was used.

### Location of SeNPs within the *B. diminuta* cells

Through investigation utilizing transmission electron microscopy (TEM), the location of SeNPs within the bacterial cells was ascertained. After centrifugation at 6000 rpm for 6 min, the bacterial cultures produced in the optimized media were collected. After collecting the pellet, it was washed with normal saline. The cells were incubated at 4 °C for an entire night in phosphate buffered saline (PBS) after being fixed with 2.5% gluteraldehyde in 0.1 M phosphate buffer (pH 7.4). Cells were dehydrated in increasing grades of ethanol (70, 90, 96, and 100%) following their embedding in agar and post-fixation in 2% osmium tetroxide. After that, EPON 812 epoxy was used to embed the cells. Using a diamond knife, they were thin-sliced to a maximum thickness of 80–100 nm. Both uranyl acetate and lead citrate were used to stain the sections.

### Bioelectrochemical characterization of *B. diminuta* consortium

#### Construction and operation of single chamber MFC

Four identical SCMFCs with a working volume of 100 mL were utilized, as previously mentioned [30]. The carbon felt anodes were three-dimensional with an estimated active surface area of 18.50 cm^2^. The cathodes were made of a non-wet proof carbon cloth with a microporous layer (projected active surface area was 16.6 cm^2^). Titanium wires were employed as current collectors between the anode and cathode electrodes. Two SCMFCs were inoculated with isolated *B. diminuta* consortium and operated separately in batch mode with synthetic media (NB contains 3.5 g L^− 1^ sodium acetate as electron donor) and real wastewater (Zenien wastewater treatment plant, Bolaq Aldakrur, Giza, Egypt) both MFCs containing 350 mg L^− 1^ of Na_2_SeO_3_, respectively. To assess the Na_2_SeO_3_ reduction capability of the biomass, control experiments were performed using synthetic media (NB contains 3.5 g L^− 1^ sodium acetate) and real wastewater with Na_2_SeO_3_-free medium. Once, the voltage output was reset to less than 0.05 V, the medium solution was refreshed. At 30 ± 2 ^o^C, each experiment was carried out in triplicate.

#### Electrochemical measurements and analysis

The potential of the SCMFCs was monitored and recorded every 5 min, using a data acquisition system (Lab Jack U6-PRO, USA). Using a resistor box (Voltcraft R-BOX 01, China), the external resistors were changed from 100 kΩ to 50 Ω in reducing order stepwise to obtain power and polarization graphs. Furthermore, the coulombic efficiency (CE), power density, and current density were estimated in accordance with other descriptions [8, 17]. In addition, the analysis of chemical oxygen demand (COD) followed the guidelines provided in Standards Methods for Water and Wastewater Examination. Furthermore, cyclic voltammetry (CV) was performed by Gamry workstation (Interface 1010E, Germany) with an electrochemical electrode placed parallel to each other. The anode served as the working electrode, and the air cathode and Ag/AgCl electrode as the auxiliary and reference electrodes, respectively. CV was measured in the potential window of − 0.8 to 0.8 V vs. Ag/AgCl at a scan rate of 5 mV s^− 1^. Additionally, electrochemical impedance spectroscopy (EIS) of the cathodes and anode was used to perform the electrochemical analyses of the SCMFCs via electrochemical workstation (Gamry, Interface 1010E, Germany) with a scanning frequency range of 100–0.001 kHz and 10 points per decade at open circuit voltage.

#### Scanning electron microscopy-energy dispersive X-ray (SEM-EDX) analysis

The morphology and elemental composition of the bioanode and biocathode were examined via SEM (SEM Quanta FEG 250 with field emission gun, FEI Company, Netherlands). The biofilms were fixed in 0.1 M phosphate buffer (pH 7.0) with 2.5% (v/v) glutaraldehyde for overnight [35]. Dehydration was induced by using ethanol gradients of 25, 50, 70, and 100%. The biofilm comprising carbon felt and carbon cloth was sputter-coated with gold and examined at a voltage of 20 kV after being let dry at 30 ^o^C. Furthermore, the elemental composition was established using EDX analysis.

## Results and discussion

The simultaneous removal of Na_2_SeO_3_ by *B. diminuta* consortium in SCMFC treating synthetic media polluted with Na_2_SeO_3_ or real wastewater was demonstrated for the first time in this work. Electroactive *B. diminuta* was employed in the SCMFC design to accomplish the oxidation of organic matter and the reduction of soluble Se oxyanions to their corresponding elemental nanoparticle form.

### Screening and identification of selenite reducing bacteria

**Fig. 1:**
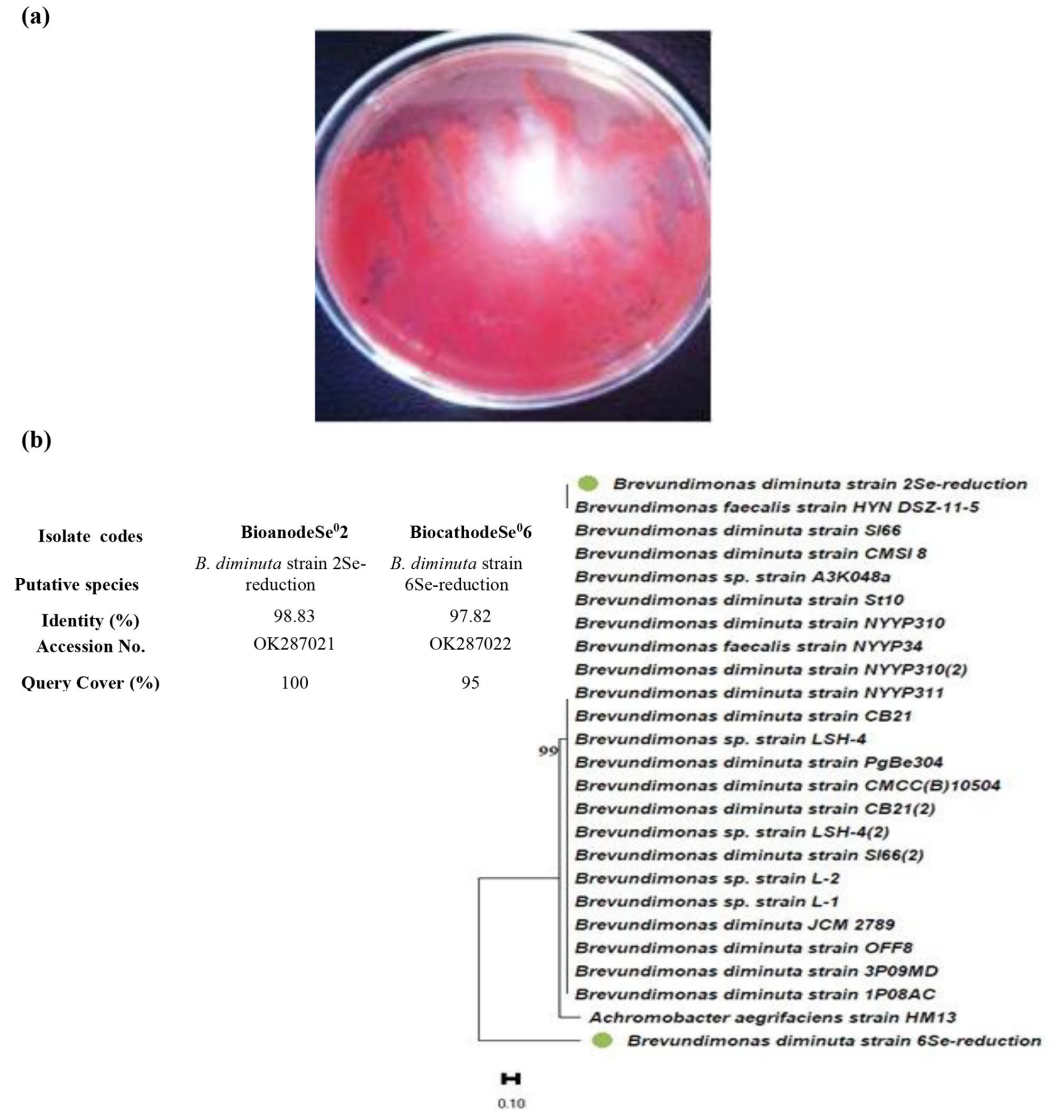
Growth of *B. diminuta* in nutrient agar in the presence of selenite **(a)** turned to red after culturing for 48 h. **(b)** Neighbor-Joining (NJ) tree of two isolated bacteria with closest relatives, inferred using MEGA software version 11. Bootstrap values expressed as percentage of 1000 replications were given at the nodes. Bar equals 0.10% sequence variation. The newly isolated strains were in circle green

From SCMFC that had previously been inoculated with activated sludge, the electroactive *B. diminuta* that reduced selenite was isolated. Both anodic and cathodic biofilms’ surfaces were scraped in order to isolate the microbial communities. Using NA plates enriched with 300 mg L^− 1^ Na_2_SeO_3_, twelve distinct bacterial strains were cultivated. These monocultures, designated bioanode 2 Se^0^ (isolates #02) and biocathode 6Se^0^ (#06), were selected due to their capacity to reduce selenite into red elemental Se^0^ and their tolerance rate towards varying doses of Na_2_SeO_3_ (Fig. 1a).

16 S rRNA regions were sequenced in order to diagnose the strains of bioanode 2Se^0^ and biocathode 6Se^0^. OK287021 and OK287022 were the accession numbers for the sequences that had been deposited in the GenBank database. Using BLAST to identify regions of similarity between biological sequences, it was determined that both strains (bioanode 2Se^0^ and biocathode 6Se^0^) were most closely related to *B. diminuta* and should be placed in the genus *Brevundimonas*. According to the results of the phylogenetic analysis, the two isolated strains were belonged to the Alphaproteobacteria class’s Caulobacterales order as well. Strain bioanode 2Se^0^ was affiliated with the *B. diminuta* cluster of the phylogenetic tree and its closest relative was *B. diminuta*, which shared 98.83% sequence similarity. Whereas, biocathode 6Se^0^ strain was affiliated to a separate branch within the Caulobacteraceae family, as confirmed by a bootstrap value of 97.82% (Fig. 1b).

The *B. diminuta* has a wide range of biological activity and is found in both terrestrial and aquatic habitats [4]. It was isolated from arsenic-polluted soil and eliminate arsenic polluted soil [36]. Additionally, it could be found in mining soils, which can be exploited to bioremediate harmful metal contamination [37]. Furthermore, it may function as an ammonia-oxidizing bacteria and an economical, green copper bioremediation agent [38, 39]. *B. diminuta* is also existing in mixed bacteria when studying the electricity-generation performances of the mixed bacteria microbial fuel cell [40]. This was the first time that these electroactive *B. diminuta* had been isolated and studied for their potential to reduce selenite and biosynthesize elemental SeNPs. Hence, these newly isolated strains were viable candidates for application in removal and recovery of selenium from contaminated selenium wastewater, so as to be utilized for the bioremediation related investigations.

### Central composite design analysis

To comprehend the efficacy of the treatment, the growth, COD, and selenite reduction were investigated. Carbon, energy, and electron donors were needed for bacteria to effectively remove selenite. Acetate was added to the broth as an electron donor to aid in the reduction of selenium, and additional nutrients were used to support the development of bacteria. In this work, we optimized the acetate concentrations and inoculum level to maximize Na_2_SeO_3_ reduction and prevent secondary contamination from excess nutrients. The most important variables that affect bacterial biomass, selenium reduction, and COD removal were selenite concentration (A), sodium acetate (B), inoculum size of bioanode 2Se^0^ strain (C), and inoculum size of biocathode 6Se^0^ strain (D). Using statistically set trials, experiments were conducted for various combinations to investigate the combined influence of these variables. The results obtained for bacterial biomass (Y1), selenite reduction (Y2), and COD removal (Y3) of actual and predicted values are listed in Table 1.

Analysis showed that with increasing the bacterial inoculation percentage from 1 to 5%, acetate from 0.5 to 6.5 g L^− 1^, and the selenite concentration from 50 to 650 mg L^− 1^ under optimal conditions, all responses were changed (Table 1). A simultaneous decrease in selenite content indicated that selenite was converted to elemental Se^0^ throughout the bacterial consortium’s development and the growth biomass of the consortium increased to a maximum value (15.38 Log CFU mL^− 1^) and then decreased slightly. While the growth rate was affected by varying acetate concentrations, a dose of 3.5 g L^− 1^ increased the biomass of cells. The consortium removed selenite, in correlation with the increasing biomass of the culture and COD removal. The efficiency of selenite removal might be improved by providing smaller amounts of electron donors. This could result in a reduction of the organic supply needed for selenite bioremediation. The initial selenite concentration increased in correlation with the decrease in selenite removal efficiency. In the presence of the selenite concentration (350 mg L^− 1^), conversion of SeO_3_^2−^ to Se^0^ was found, indicating bacterial growth and the functioning of metabolic conversions.

The bacterial biomasses were between 9.01 and 15.38 Log CFU mL^− 1^; COD removal varied between 75.60% and 89.94%, and selenium reduction were between 91.05% and 99.08%. The following Eqs. (1–3) represent the quadratic equations that were obtained:

1 $$\begin{gathered}Y1 = - 17.46495 + 0.047082A + 3.59046B \hfill \\\,\,\,\,\,\,\,\,\,\, + 6.66875C + 5.15208D - 0.000397AB \hfill \\\,\,\,\,\,\,\,\,\,\, - 0.000679AC + 0.001671AD - 0.225417BC \hfill \\\,\,\,\,\,\,\,\,\,\, + 0.156250BD - 0.638125CD - 0.000067A \hfill \\\,\,\,\,\,\,\,\,\,\, - 0.461713B - 0.577604C - 0.741354D \hfill \\ \end{gathered}$$

2 $$\begin{gathered}Y2 = + {\text{ }}48.78048 - 0.026107A + 14.85781B \hfill \\\,\,\,\,\,\,\,\,\,\,\,\, + 4.83630C + 4.24618D + 0.002778AB \hfill \\\,\,\,\,\,\,\,\,\,\,\, + 0.005442AC + 0.003450AD - 0.438333BC \hfill \\\,\,\,\,\,\,\,\,\,\,\, - 0.379167BD + 0.232500CD - 0.000018A \hfill \\\,\,\,\,\,\,\,\,\,\,\, - 1.52241B - 0.884453C - 0.713024{D^2} \hfill \\ \end{gathered}$$

3 $$\begin{gathered}Y3 = + 69.46986 + 0.046319A + 1.62361B \hfill \\\,\,\,\,\,\,\,\,\,\,\, + 7.84625C + 3.56208D - 0.000819AB \hfill \\\,\,\,\,\,\,\,\,\,\,\, + 0.003796AC - 0.003262AD + 0.010417BC \hfill \\\,\,\,\,\,\,\,\,\,\,\, + 0.109583BD - 0.024375CD - 0.000048A \hfill \\\,\,\,\,\,\,\,\,\,\,\, - 0.241250B - 1.66031C - 0.366562{\text{ }}{D^2} \hfill \\ \end{gathered}$$

where Y1, Y2, and Y3 were the predicted responses of Log CFU mL^− 1^, COD removal %, and Se removal %, respectively. A, B, C and D represented the coded test variables for selenite concentration (mg L^− 1^), sodium acetate (g L^− 1^), inoculum size of bioanode 2Se^0^ (%), and inoculum size of biocathode 6Se^0^ (%), respectively. In order to investigate parameter hypotheses, the ANOVA statistical approach divided the total variations into component parts linked to certain sources of variation (Table 2**)**. The growth, COD removal, and selenium removal were significantly correlated with selenite concentration, sodium acetate, inoculum size of bioanode 2Se^0^, inoculum size of biocathode 6Se^0^, quadratic coefficients (A^2^, B^2^, C^2^ and D^2^), and interaction coefficient (AB, AC, AD, and BD). Large model F-values (401.12, 255.21, and 155.21) indicated that the regression equations represented the majority of the variation in all responses, indicating the significance of the constructed quadratic models in predicting Log CFU mL^− 1^, COD removal, and selenium removal. Based on the R^2^ value, which indicated the relevance of the models for all responses, a well-fitted link between the experimental and predicted response values was established. As shown in Table 2, the adjusted R^2^ of 0.9948, which denoted an adequate signal, and the Log CFU mL^− 1^ predicted R^2^ of 0.9847 matched reasonably well.

**Table 1:** The experimental runs for the factors and the observed responses through CCD.

**Run order**	A: **Selenite conc.** **(mg L** ^**− 1**^ **)**	B: **Sodium acetate (electron donor)** (g L^− 1^)	C: Inoculum size of anodic bacterial isolate (%)	D: Inoculum size of cathodic bacterial isolate (%)	Log CFU mL^− 1^, R1		COD removal (%), R2		Se removal (%), R3	
					Actual value	Predicted value	Actual value	Predicted value	Actual value	Predicted value
1	500	5	2	2	10.81	10.83	88.32	87.89	96.90	96.95
2	350	3.5	3	3	15.38	15.38	89.94	89.94	99.08	99.08
3	350	3.5	3	1	12.73	12.57	85.60	86	96.53	96.55
4	500	2	4	2	13.06	13.04	79.20	79.7	96.97	97.17
5	50	3.5	3	3	9.01	8.83	88.57	88.98	91.05	91.35
6	500	2	2	2	10.6	10.76	75.60	76.03	97.73	97.66
7	350	3.5	3	3	15.38	15.38	89.94	89.94	99.08	99.08
8	350	3.5	3	3	15.38	15.38	89.94	89.94	99.08	99.08
9	350	3.5	3	3	15.38	15.38	89.94	89.94	99.08	99.08
10	200	2	2	2	10.38	10.38	81.20	80.58	94.34	94.06
11	350	3.5	3	3	15.38	15.38	89.94	89.94	99.08	99.08
12	500	5	2	4	12.88	12.92	88.16	88.42	97.35	97.42
13	500	5	4	2	11.65	11.75	89.44	88.94	96.71	96.53
14	200	5	4	2	12.05	12.13	88.32	87.73	91.38	91.39
15	350	6.5	3	3	11.42	11.26	83.89	84.4	96.98	96.93
16	350	3.5	1	3	12.69	12.55	85.60	85.21	93.96	94.09
17	350	3.5	3	3	15.38	15.38	89.94	89.94	99.08	99.08
18	350	0.5	3	3	11.3	11.19	68.58	68.08	96.55	96.88
19	200	2	4	4	10.57	10.65	82.28	82.65	93.16	92.96
20	500	2	2	4	11.89	11.91	78.29	78.83	97.62	97.46
21	350	3.5	3	5	12.37	12.26	88.57	88.18	98.41	98.68
22	650	3.5	3	3	9.94	9.85	88.11	87.71	98.14	98.12
23	200	2	2	4	10.45	10.52	80.76	81.31	95.78	95.83
24	200	5	4	4	10.65	10.66	87.49	87.11	93.78	93.71
25	350	3.5	5	3	13.72	13.59	87.20	87.6	90.63	90.79
26	200	5	2	2	10.67	10.8	89.33	89.94	93.95	94.1
27	200	2	4	2	12.92	13.06	81.20	80.99	91.5	91.3
28	500	5	4	4	11.18	11.28	89.83	90.39	96.76	96.89
29	200	5	2	4	11.76	11.88	88.96	88.4	96.86	96.52
30	500	2	4	4	11.59	11.63	84.00	83.43	97.16	96.87

**Table 2:** ANOVA results for the quadratic model with responses

Source	Log CFU/mL					COD removal (%)					Selenite removal (%)				
	Sum of Squares	df	Mean Square	F-value	*P-value*	Sum of Squares	df	Mean Square	F-value	*P-*value	Sum of Squares	df	Mean Square	F-value	*P-*value
**Model**	101.76	14	7.27	401.12	< 0.0001	779.8	14	55.7	155.21	< 0.0001	198.74	14	14.2	255.21	< 0.0001
**Linear**															
**A**	1.54	1	1.54	84.72	< 0.0001	2.42	1	2.42	6.73	0.0203	68.78	1	68.78	1236.6	< 0.0001
**B**	0.0077	1	0.0077	0.4252	0.5242	399.71	1	399.71	1113.82	< 0.0001	0.0035	1	0.0035	0.063	0.8052
**C**	1.65	1	1.65	90.97	< 0.0001	8.57	1	8.57	23.88	0.0002	16.29	1	16.29	292.79	< 0.0001
**D**	0.1488	1	0.1488	8.21	0.0118	7.15	1	7.15	19.93	0.0005	6.77	1	6.77	121.77	< 0.0001
**Interaction**															
**AB**	0.1278	1	0.1278	7.05	0.018	6.25	1	6.25	17.42	0.0008	0.5439	1	0.5439	9.78	0.0069
**AC**	0.1661	1	0.1661	9.16	0.0085	10.66	1	10.66	29.71	< 0.0001	5.19	1	5.19	93.25	< 0.0001
**AD**	1.01	1	1.01	55.46	< 0.0001	4.28	1	4.28	11.94	0.0035	3.83	1	3.83	68.89	< 0.0001
**BC**	1.83	1	1.83	100.95	< 0.0001	6.92	1	6.92	19.27	0.0005	0.0039	1	0.0039	0.0702	0.7946
**BD**	0.8789	1	0.8789	48.5	< 0.0001	5.18	1	5.18	14.42	0.0018	0.4323	1	0.4323	7.77	0.0138
**CD**	6.52	1	6.52	359.55	< 0.0001	0.8649	1	0.8649	2.41	0.1414	0.0095	1	0.0095	0.1709	0.6852
**Quadratic**															
**A²**	62.55	1	62.55	3451.8	< 0.0001	4.36	1	4.36	12.15	0.0033	32.31	1	32.31	580.84	< 0.0001
**B²**	29.6	1	29.6	1633.58	< 0.0001	321.83	1	321.83	896.8	< 0.0001	8.08	1	8.08	145.29	< 0.0001
**C²**	9.15	1	9.15	505	< 0.0001	21.46	1	21.46	59.79	< 0.0001	75.61	1	75.61	1359.35	< 0.0001
**D²**	15.07	1	15.07	831.92	< 0.0001	13.94	1	13.94	38.86	< 0.0001	3.69	1	3.69	66.26	< 0.0001
**Residual**	0.2718	15	0.0181			5.38	15	0.3589			0.8343	15	0.0556		
**Lack of Fit**	0.2718	10	0.0272			5.38	10	0.5383			0.8343	10	0.0834		
**Pure Error**	0	5	0			0	5	0			0	5	0		
**Cor Total**	102.03	29				785.19	29				199.57	29			
**R** ^**2**^				0.9973					0.9931					0.9958	
**Adj. R** ^**2**^				0.9948					0.9867					0.9919	
**Pred R** ^**2**^				0.9847					0.9605					0.9759	

A: selenite conc. (mg L^− 1^); B: sodium acetate (electron donor) (g L^− 1^); C: inoculum size of anodic bacterial isolate (%); D: inoculum size of cathodic bacterial isolate (%); Df: degree of freedom

**Fig. 2:**
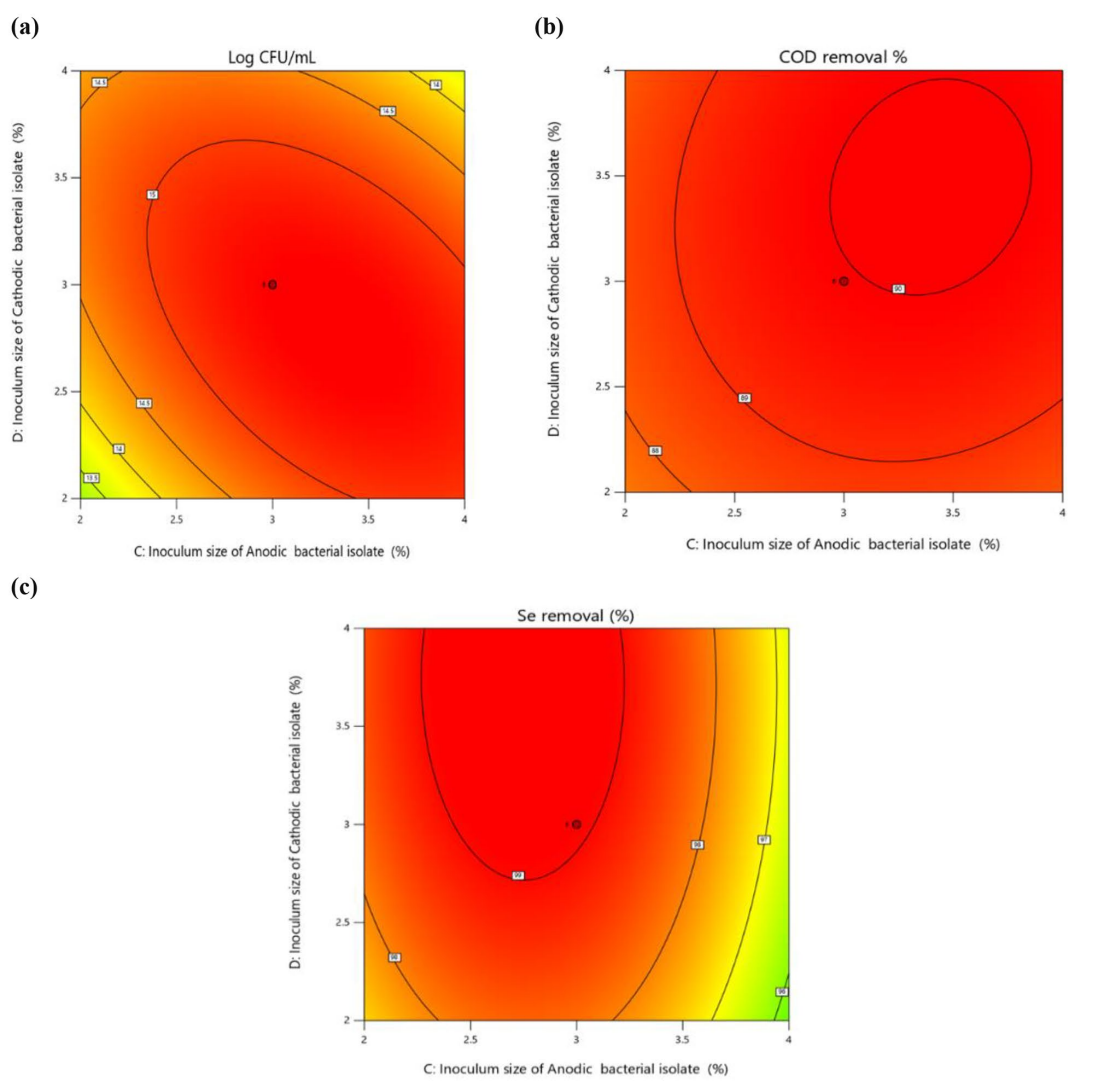
2D contour plots for Log CFU mL^− 1^ **(a)** COD removal (%), **(b)** and Se removal (%), **(c)** using data in Table 1. Inputs, 30 experimental runs carried out under conditions established by CCD

Figure 2(a-c) and Fig. S1 display 2D contour plots that illustrated the complex relationship between different variables on Log CFU mL^− 1^, COD removal and selenium removal. Two variables with fixed values at their control level were shown interacting in the contour plots. When the inoculation amount increased from 1 to 3%, the cell biomass and COD and selenium removal were improved. However, all responses were decreased when the inoculation dosage was increased further to 4%. The trend in responses variations was similar for both sodium acetate and selenite concentration increases. This outcome might be explained by the possibility that the growth of the consortium was impeded when the inoculation level, selenite concentration, and sodium acetate exceeded a particular range. The reason for this was the biological molecules’ binding sites becoming saturated, which results in the reduction of SeO_3_^2−^ into SeNPs [41]. When compared to lower and medium inoculum sizes, *B. diminuta* produced noticeably more SeNPs at higher inoculum sizes because the solution contained more reducing molecules. Based on the results of the experiments and the model analysis, the maximum growth, COD and selenium removal of the consortium (15.38 Log CFU mL^− 1^, 89.94%, 99.08%, respectively) could be achieved under the optimal conditions: 350 mg L^− 1^ selenite concentration, 3.5 g L^− 1^ sodium acetate, 3% inoculum size of bioanode 2Se^0^, and 3% inoculum size of biocathode 6Se^0^.

It was evident that the initial selenite concentration and/or the bacterial inoculum level were likely connected to the selenite reduction rate and efficiency for *B. diminuta*. It was also plausible that cellular reductases and/or reducing substances, whose synthesis and consumption were associated with the microbe’s growth phase, mediate selenite reduction [42]. The transportation and destiny of microbial generated nanoparticles in the environment were influenced by the organic material that surrounds them [43]. To allow biological treatment, the inorganic wastewater could be supplemented with carbon substrate and electron donor. Numerous forms of carbon sources, such as acetate, have been studied [44]. The selenite’s ability to prevent bacterial growth resulted in a decrease in selenite reduction at greater doses. *Bacillus paramycoides* was previously found to exhibit delayed reduction of SeO_3_^2−^ in the presence of greater concentrations of SeO_3_^2−^ [45].

Microbial reduction is a workable method for recovering Se for material sustainability, treating wastewater, and bioremediation [27]. It takes less energy and electrons to reduce selenite [46]. The assembly and stabilization of SeNPs by microorganisms are controlled by a number of variables, including the concentration of biomass and selenium oxyanion [47]. Depending on the original wastewater, different bacterial strains would be used. If the wastewater did not contain organic carbon sources, it would be best to provide the treatment system with simple organic carbons like acetate, and in this case, owing to their greater rates of selenite reduction, *B. diminuta* strain 2Se-reduction and *B. diminuta* strain 6Se-reduction would be the more optimal choices. Waste-based carbon sources, including wastewater, could be investigated for use as carbon sources for microbial selenite reduction to elemental selenium in SCMFC as a sustainable approach.

### Analytical characterization of SeNPs produced by *B. diminuta* consortium

#### UV–vis spectroscopy

**Fig. 3:**
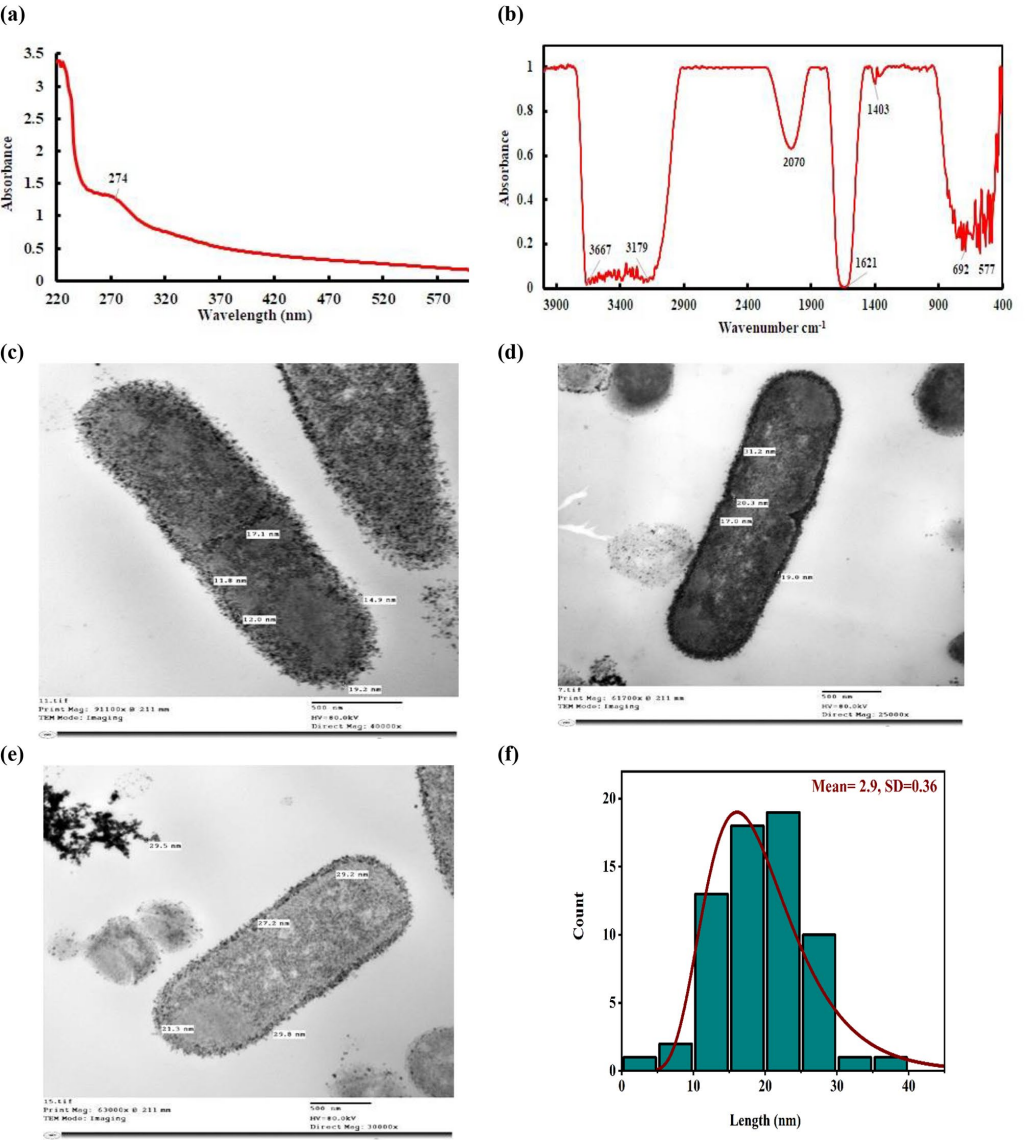
Characterization of SeNPs produced from consortium *(B. diminuta* strain 2Se-reduction and *B. diminuta* strain 6Se-reduction) cultivated with Na_2_SeO_3_ at 37 ^o^C for 48 h. **(a)** UV and **(b)** FTIR of SeNPs. **(c-e)** TEM images of localization of SeNPs. **(f)** Size distribution of the SeNPs formed in the scale bar of 500 nm

Using a consortium of *Brevundimonas*, the UV-Vis spectrum was used to confirm the production of SeNPs, suggesting that Na_2_SeO_3_ might be physiologically reduced to the elemental Se^0^. A bright red color was exhibited by Bio-Se^0^ that resulted from SeO_3_^2−^ reduction culture. The analysis showed that SeNPs led surface plasmon resonance (SPR) vibration effects around 271 nm (Fig. 3a). The wide SPR peak clearly displays the polydispersity of the SeNPs [48]. According to [49], if the particle size was 100 nm or less, it demonstrated a distinct absorption maximum in the UV range.

Our research validated the findings of Zhang et al. [50], who found an absorption peak associated with SeNPs at 200–300 nm. Wen et al. [2] reported that a wavelength shift suggests a larger particle size and an increase in the absorption peak indicates a higher number of particles in the mixture. This is because, in order to induce plasma resonance, smaller particles with higher surface energy need more energetic light with a shorter wavelength, whereas larger particles operate oppositely [51]. The reduction of Na_2_SeO_3_ to SeNPs by biomolecules produced by the *B. diminuta* acted as reducing agents.

#### FTIR analysis

Using the infrared spectrum, the functional groups of the bacterial biomolecules serving as capping and reducing agents during the nanoparticle synthesis were determined (Fig. 3b). Two prominent peaks were visible in the FT-IR spectra of SeNP. The first peak, which represented the –OH and NH, had a wide range from 3667 cm^− 1^ to 3179 cm^− 1^. The second peak was 1621 cm^− 1^, which connected to –NH stretches, suggesting the presence of carboxylic and amide groups. Because the amide I band mostly overlapped the asymmetric counterpart, which could be observed as a peak at 1621 cm^− 1^, the band at 1403 cm^− 1^ might be attributed to the symmetric stretching vibrations of carboxylate (COO-) [34]. In the process of turning Na_2_SeO_3_ into Se^0^, these protein functional groups enabled the reduction and stabilization of SeNPs. This was reported in biosynthesis of SeNPs using bacteria *Streptomyces minutiscleroticus* [52] and *Pseudomonas alcaliphila* [50]. Thus, our results not only supported the presence of bacterial proteins in selenite reduction but also the synthesis and stabilization of SeNPs by these proteins [53].

#### Location of SeNPs within consortium cells

The TEM analysis of the cultures cultivated with selenite revealed additional confirmation of the spherical internal and extracellular deposits of SeNPs (Fig. 3c-e). The spherical particles had a size range of 11.8–31.2 nm, with an average of 21.5 nm like those identified in *Duganella* sp. and *Agrobacterium* sp [54]. Our consortium might be selenite-tolerant due to an internal decrease of these SeO_3_^2−^, followed by their accumulation in the cytoplasm or periplasmic space, and finally their exudation by the bacterial cell. Additionally, a large number of tiny particles—possibly bound by extracellular polymeric substances were gradually seen on the cell surface. There was no indication of external membrane deformation or cell lysis. As illustrated in Fig. 3(f), the distribution size of SeNPs in the bacterial cells showed a size distribution spanning between 12.5 and 27.5 nm. Furthermore, the log normal distribution curve demonstrated that the distribution was perfectly fitted because the standard deviation was nearly zero (0.36).

### Anodic bioelectrogenic activity

#### Voltage output

**Fig. 4:**
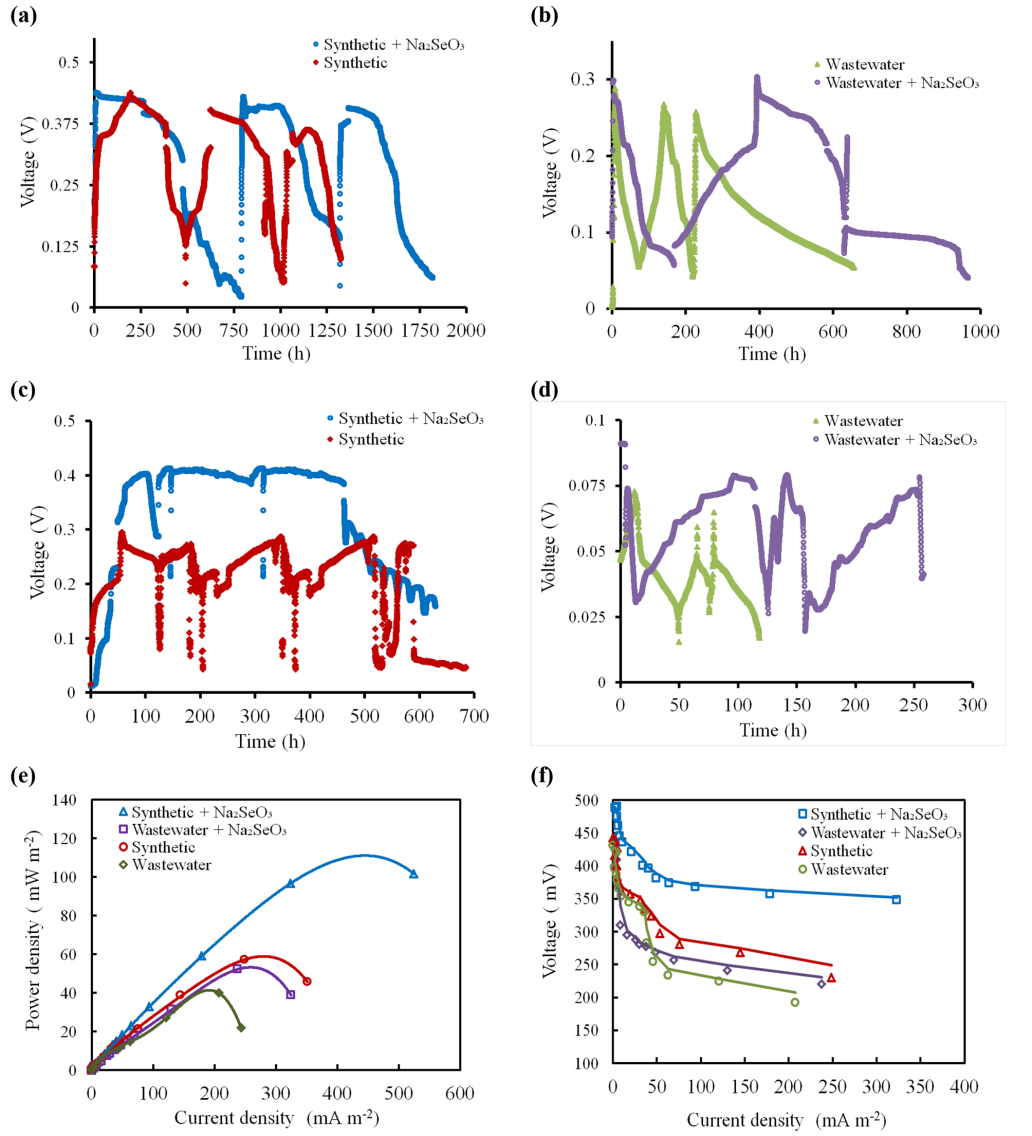
**(a, b)** Voltage vs. time (OCV) curve; (**c, d**) Voltage vs. time (CCV) curve and **(e, f)** Polarization and power curves for synthetic + Na_2_SeO_3_, synthetic, wastewater + Na_2_SeO_3_ and wastewater, respectively

Four identical air-cathode, single-chamber MFCs (SCMFCs) were used to assess the ability of *B. diminuta* toward the selenite reduction to SeNPs. Two SCMFCs were inoculated with *B. diminuta* consortium and operated separately in batch mode with synthetic media (synthetic + Na_2_SeO_3_) and real wastewater (wastewater + Na_2_SeO_3_) both MFCs containing 350 mg L^− 1^ of sodium selenite and 3.5 g L^− 1^ of Na_2_SeO_3_ as electron donor, respectively. For comparison, control experiments were performed using synthetic media (synthetic) or real wastewater (wastewater) without sodium selenite. Figure (4) illustrates the change of open circuit voltages (OCVs) and close circuit voltages (CCVs) with time as a function of increasing the biofilm electrogenic performance with respect to the reducing of selenite. It could be observed from Fig. 4 (a, b) that the maximum and steady-state OCV in synthetic + Na_2_SeO_3_ was 0.442 ± 0.04 mV, followed by synthetic (400 ± 0.04 mV), wastewater + Na_2_SeO_3_ (340 ± 0.09 mV) and wastewater (284 ± 0.06 mV). Thus, the OCVs results might be attributed to the successful development of *B. diminuta* consortium on the surface of anodic graphite felt forming active biofilm and consequently the SCMFCs takes shorter periods to decrease the startup time for electricity generation [15]. Additionally, Fig. 4 (c, d) demonstrates the effect of 1 KΩ on the performance of SCMFCs voltage outputs. For instance, the CCVs for synthetic + Na_2_SeO_3_ and synthetic SCMFCs were 409 ± 0.006 and 275 ± 0.001 mV, respectively, which were significantly higher than that of other SCMFCs (i.e., 78 ± 0.005 mV for wastewater + Na_2_SeO_3_ and 62 ± 0.008 mV for wastewater), confirming that the addition of Na_2_SeO_3_ had better influence on the performance of both synthetic and wastewater SCMFCs towards bioelectricity generation than the compared control for each reactor.

#### Polarization and power density

Power and polarization curves were measured at a steady-state OCV for all the tested SCMFCs using different external loads from 100 kΩ to 50 Ω (Fig. 4e, f). The maximum power density was observed in synthetic + Na_2_SeO_3_ (101.72 mWm^− 2^), followed by synthetic (57.18 mWm^− 2^), wastewater + Na_2_SeO_3_ (52.33 mWm^− 2^) and wastewater (39.85 mWm^− 2^) at cell current densities of 524.32, 248.65, 237.84, and 207.57 mAm^− 2^ for synthetic + Na_2_SeO_3_, synthetic, wastewater + Na_2_SeO_3_, and wastewater, correspondingly (Fig. 4e). Moreover, the cell design point for all tested SCMFCs was observed at 100 Ω. Besides, the internal resistance of all SCMFCs was estimated by performing linear regression analysis of the ohmic zone in polarization plots (Fig. 4f). Between all SCMFCs, the synthetic + Na_2_SeO_3_ revealed the lowest internal resistance of 264 Ω, followed by 337 Ω, 441 Ω and 530 Ω for synthetic, wastewater + Na_2_SeO_3_ and wastewater. Consequently, the addition of Na_2_SeO_3_ enhance the performance of electrogenic *B. diminuta* in SCMFCs that contain synthetic or wastewater towards bioelectricity generation and decreases the internal resistance leading to a stable operating system with reducing SCMFCs losses [13]. The coulombic efficiencies (CE) of all SCMFCs were estimated to be 31.7 ± 0.064%, 19 ± 0.043%, 7.96 ± 0.066% and 3.84 ± 0.024% for synthetic + Na_2_SeO_3_, synthetic, wastewater + Na_2_SeO_3_ and wastewater, respectively. The CE might be attributed to the addition of Na_2_SeO_3_ [8, 22].

#### Cyclic voltammetry (CV)

**Fig. 5:**
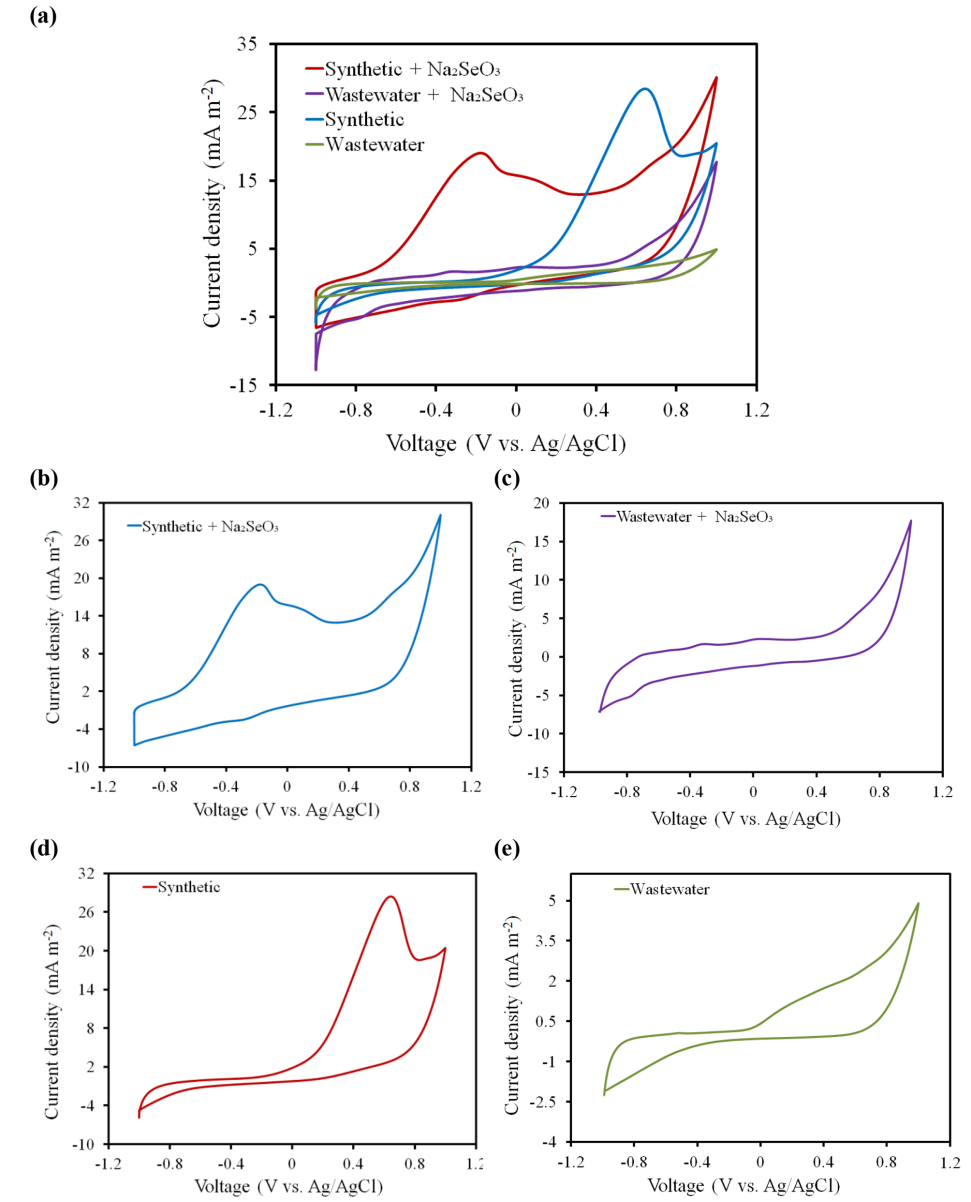
**(a)** Cyclic voltammetry of the *B. diminuta* consortium behavior towards the selenite reduction for all SCMFCs bioreactors, (**b-e)** cyclic voltammetry of synthetic + Na_2_SeO_3,_ wastewater + Na_2_SeO_3_, synthetic and wastewater SCMFCs, respectively, at a scan rate of 5 mVs^− 1^ over the potential window from – 1.0 V to 1.0 V vs. Ag/AgCl

The bioelectrocatalytic behavior of the *B. diminuta* towards the selenite reduction in all SCMFCs bioreactors was tested at steady state OCV and elucidated in situ through CV over the potential window from – 1.0 to 1.0 V vs. Ag/AgCl reference electrode at a scan rate of 5 mV s^-1^. As clarified in Fig. 5(a), there were different patterns of CV curves, depending on the presence and absence of Na_2_SeO_3_. Remarkably, synthetic + Na_2_SeO_3_ revealed two oxidative peaks at -0.172 V and 0.106 V beside one reductive peak at -0.254 V. Also, wastewater + Na_2_SeO_3_ showed two oxidative peaks at -0.305 V and 0.063 V with one reductive peak at -0.762 V (Fig. 5b, c). Whereas, there was only one oxidative peak at 0.623 V and 0.362 V for control SCMFCs (synthetic and wastewater, respectively) as displayed in Fig. 5(d, e). The existence of reduction peaks was possibly corresponded to the formal potential that involved in the biological reduction of selenite to selenium under anaerobic condition. In addition, the lowering potential values to more negative might be related to the biological conversion of elemental selenium to selenide that was close to the theoretical redox potential at neutral pH (Eq. 4) [55]. Thus, these redox peaks revealed the secretion of a definite extracellular redox mediator by the *B. diminuta* consortium that might be involved in the electron transfer progression and hence accelerated the reduction of selenite [40, 56]. These results inferred that the selenite was expected to be reduced intracellularly into Se^0^ nanoparticles in the cell. Consequently, it was transferred extracellularly under anaerobic conditions or vice versa [57]. As mentioned earlier by Catal et al., the selenite was observed to be reduced to Se^0^ in a SCMFC [22]. However, this study also indicated selenite was not a terminal electron acceptor at the cathode, but instead may be heterotrophically reduced by anode respiring bacteria. Recently, another study investigated the non-external circuit bioelectrochemical system without both external electrical circuit and ion exchange membrane and inoculated *Shewanella sp.* HN-41 as anodic strain with the lactate substrates. They showed that the clean Se^0^ nanoparticles were synthesized and completely separated from bacterial cells in the bioelectrochemical system at the more negative potentials [58]. Generally, the biological selenite reduction by *B. diminuta* consortium probably occurred by the preliminary reduction of SeO_3_^2-^ to Se^0^ under anaerobic conditions (Eq. 4), followed by the reduction of orange- red colored Se^0^ nanoparticles to Se^2-^ (Eq. 5). Selenide is oxygen sensitive and can be re-oxidized to elemental Se^0^ in cathodic side (Eq. 6) [59]:

4 $$C{H_3}CO{O^-}\, + \,Se{O_3}^{2-}\, \to \,S{e^0}\, + \,{H_2}O\, + \,2C{O_2}\, + \,{H^+}$$

5 $$S{e^0} + 2{H^ + } + 2e \to {H_2}Se$$

6 $$2{H_2}Se + {O_2} \to 2S{e^0} \downarrow + 2{H_2}O$$

#### Electrochemical impedance

**Fig. 6:**
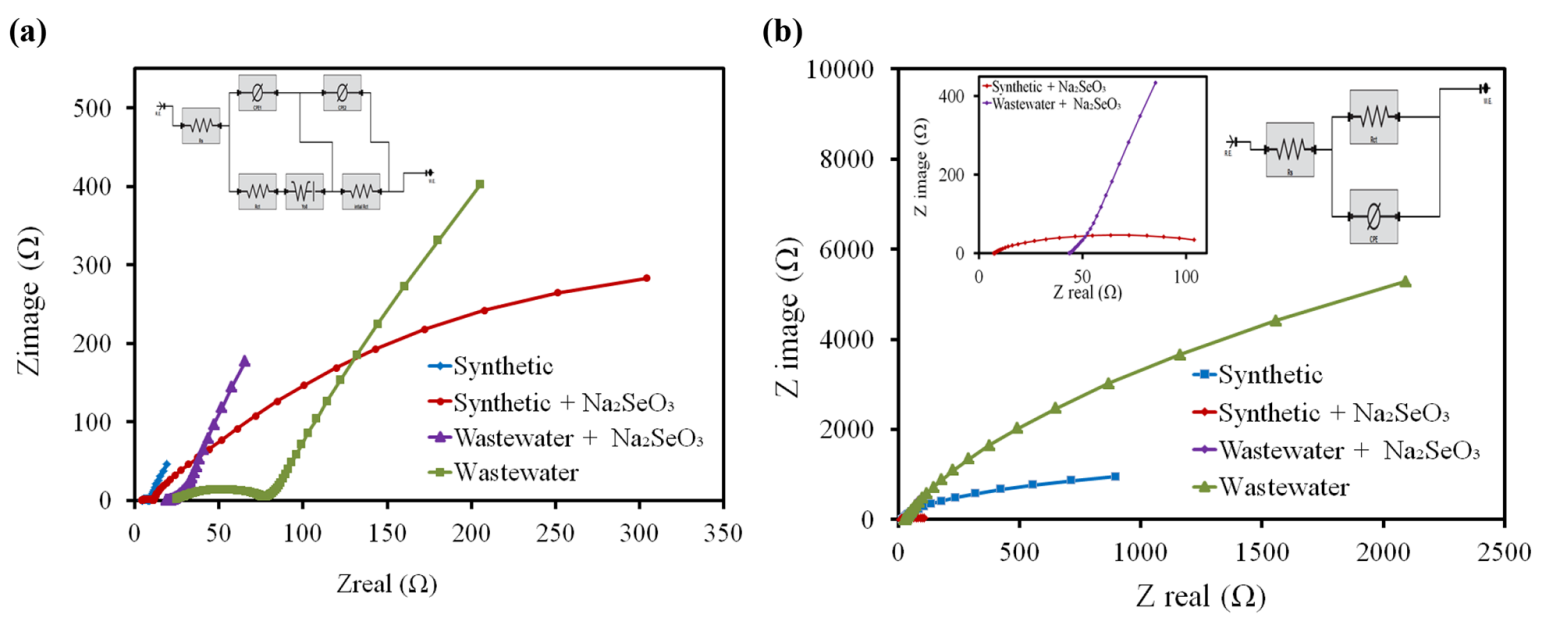
The Nyquist plots (inset picture represents the equivalent circuit used for data fitting) for (**a)** anodic and **(b)** cathode for synthetic + Na_2_SeO_3,_ wastewater + Na_2_SeO_3_, synthetic and wastewater SCMFCs

Figure 6 demonstrates the Nyquist plots and electrochemical impedance fitting for all tested bioanodes and biocathodes in all SCMFCs at steady state OCV. It could be observed that, the EIS results supported the CV results. For all tested bioanodes, it was noticed that the synthetic + Na_2_SeO_3_ (the electron transfer resistance (R_ct_) = 847.9 ± 0.13 and ohmic resistance (R_ohm_) = 3.88 ± 0.11 Ω) and wastewater + Na_2_SeO_3_ (32.27 ± 0.62 Ω and R_ohm_ = 16.53 ± 0.65 Ω) exhibited the lowest R_ct_ that were comparable with that of control SCMFCs as clarified in Table (3) and Fig. 6a. Moreover, in the case of biocathodes, the R_ct_ was lowered after the addition of Na_2_SeO_3_ than the control (Tables 3 and Fig. 6b). The lower values of R_ct_ and R_ohm_ were probably due to the capability of the *B. diminuta* consortium to produce a definite extracellular redox mediator that enhanced the kinetics of direct transfer of electrons between the *B. diminuta* and the anode [17] and enhanced electrical conductivity and ORR activity of cathodic reactions [14].

**Table 3:** The charge transfers resistance (R_ct_) and solution resistance (R_ohm_) values

**Anode EIS**	**Charge transfer resistance (** *R*_**ct**_ **) Ω**	**Solution resistance (** *R*_**ohm**_ **) Ω**
Synthetic + Na_2_SeO_3_	847.900 ± 0.13	3.88 ± 0.11
Synthetic	3885.518 ± 0.21	3.20 ± 0.18
Wastewater + Na_2_SeO_3_	32.2704 ± 0.62	16.53 ± 0.65
Wastewater	14270.73 ± 0.88	23.38 ± 0.53
**Cathode EIS **	**Charge transfer resistance (R**_**ct**_**)** Ω	**Solution resistance (R**_**ohm**_**)** Ω
Synthetic + Na_2_SeO_3_	7.4323 ± 3.08	118.36 ± 2.12
Synthetic	17.0000 ± 0.07	2593.982 ± 1.03
Wastewater + Na_2_SeO_3_	235.3700 ± 0.72	42.7912 ± 0.61
Wastewater	23713.69 ± 1.81	26.7234 ± 0.12

#### Surface morphology

**Fig. 7:**
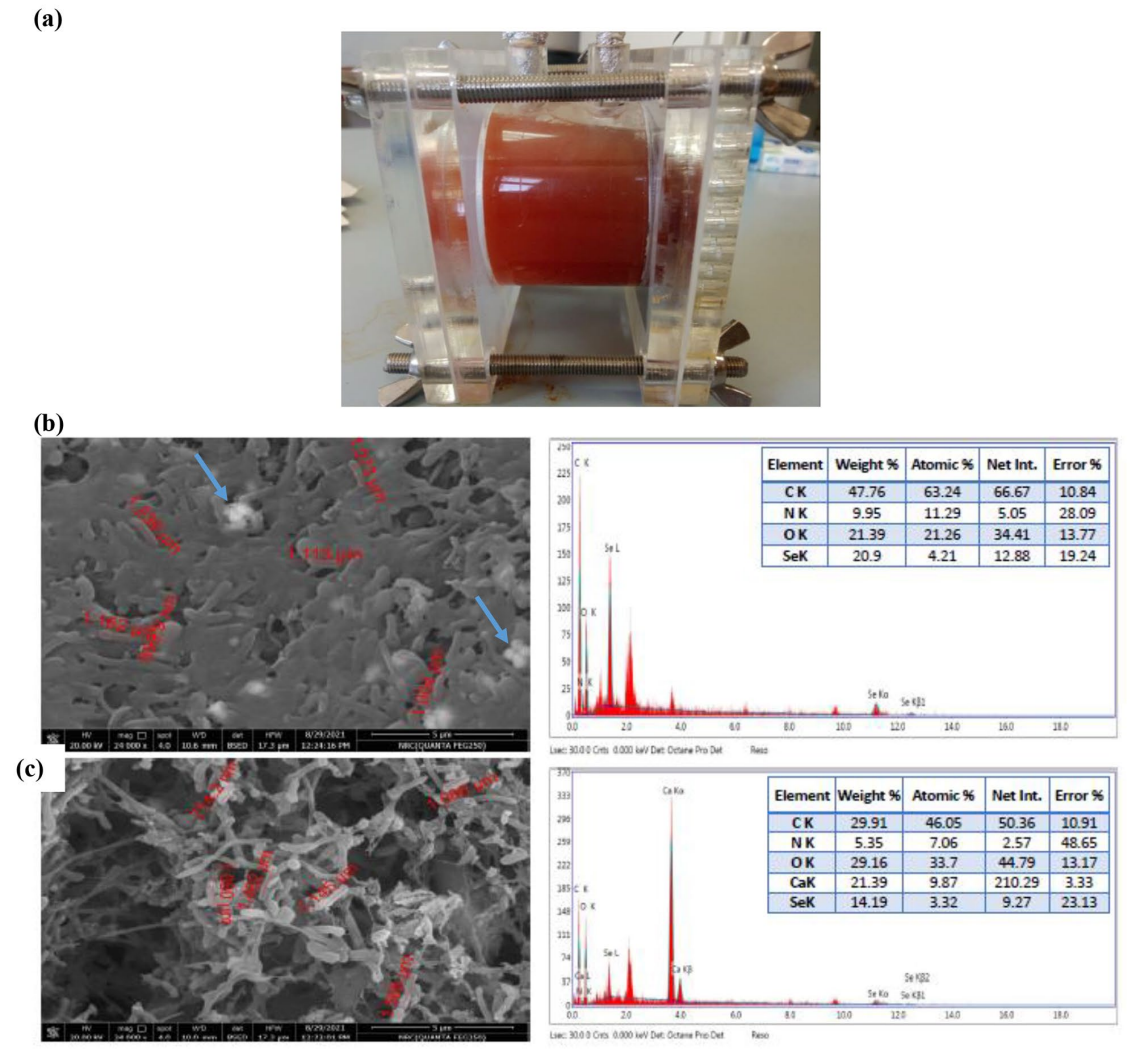
Macroscopic view of cathode and anode of MFC after fed with selenite at the end of all experiments **(a)**. SEM image of bioanode **(b)** and biocathode **(c)** and EDX analysis of the contents of carbon, nitrogen, oxygen and selenium in the area

The electrodes of SCMFC enriched with synthetic + Na_2_SeO_3_ (Fig. 7a) were examined by SEM analysis. The images revealed the bacterial cell adhesion to the anode and cathode surfaces as well as elemental SeNPs formed by the electrogenic consortium (Fig. 7b, c)). The bacteria’s sizes varied from 0.714 to 1.566 nm. Aggregates and large colonies may have contributed to the system’s improved performance in both electrodes (Fig. 7b, c). In addition, bacteria on the biocathode were adjacent to some icky-like matter (Fig. 7c), which was suspected to be extracellular polymer substances (EPS). This EPS was associated with the exoelectrogens’ capacity to adhere to the cathode. EDX analysis verified that the nanoparticles were elemental selenium, produced by the bioelectrochemical process (Fig. 7c). The elemental microanalysis spectrum of bioanode showed the presence of selenium (Se; 14.19%) along with carbon (C; 29.91%), nitrogen (N; 5.35%), oxygen (O; 29.16%), and calcium (Ca; 21.39%) (Fig. 7b). The peaks detected for the biocathode electrode deposition of Se^0^ showed the presence of selenium at 20.9%, along with the presence of C (47.76%), N (9.95%), and O (21.39%) (Fig. 7c). The presence of organic components, such as extracellular polymeric compounds or enzymes/molecules involved in the biogenesis of Se^0^ particles, is indicated by the presence of C, O, N, P, and S elements in the biofilm, which in agreement with Sudharsan et al. [60]. Reducing SeO_3_^2−^ towards Se^0^ deposition could illustrate the lowered toxicity. The most effective strategy of action for recovering and repurposing these SeNPs would be to employ this consortium for selenite bioremediation.

## Conclusion

This study was the first demonstration on electroactive *B. diminuta* consortium for bioreduction of toxic SeO_3_^2−^ to produce biogenic non-toxic orange-red colored SeNPs using SCMFC. The *B. diminuta* was capable of secretion a definite extracellular redox mediator in the presence of SeO_3_^2−^ that might be involved in the electron transfer progression and hence accelerated the bioreduction process. Moreover, the microbe-electrode and SeO_3_^2−^ interactions had a regulatory influence on the anodic microbial metabolism and its electrogenic activity, regularly, evidencing an increased enzyme activity. Thus, highly efficient transforming hazardous oxyanions into insoluble Se^0^ in SCMFC was a bioremediation strategy that aimed to treat wastewater, generate electricity and recovering rare metals simultaneously. In conclusion, *B. diminuta* could be suitable and robust bioelectrocatalyst for the application of MFCs in bioremediation plant in a highly polluted sites with oxyanions and accelerate the efficient and economical synthesis of SeNPs. The recovery and acceleration production of SeNPs rate as well as the its application in nanotechnology industries will be the main targets for our future work.

### Electronic supplementary material

Below is the link to the electronic supplementary material.

Supplementary Material 1

## Data Availability

No datasets were generated or analysed during the current study.
