# Supplementary material for: Electroactive Brevundimonas diminuta consortium mediated selenite bioreduction, biogenesis of selenium nanoparticles and bio-electricity generation
Source: J Nanobiotechnology. 2024 Jun 20;22:352. doi: 10.1186/s12951-024-02577-3 (PMC11188503; doi:10.1186/s12951-024-02577-3)
Supplement: Supplementary file 1 — Supplementary Material 1 [file 12951_2024_2577_MOESM1_ESM.docx]

**Supplementary information to article “Electroactive *Brevundimonas diminuta* consortium mediated selenite bioreduction, biogenesis of selenium nanoparticles and bio-electricity generation’’ (Sakr et al.)**

| **(a)** | | | |
| --- | --- | --- | --- |
| **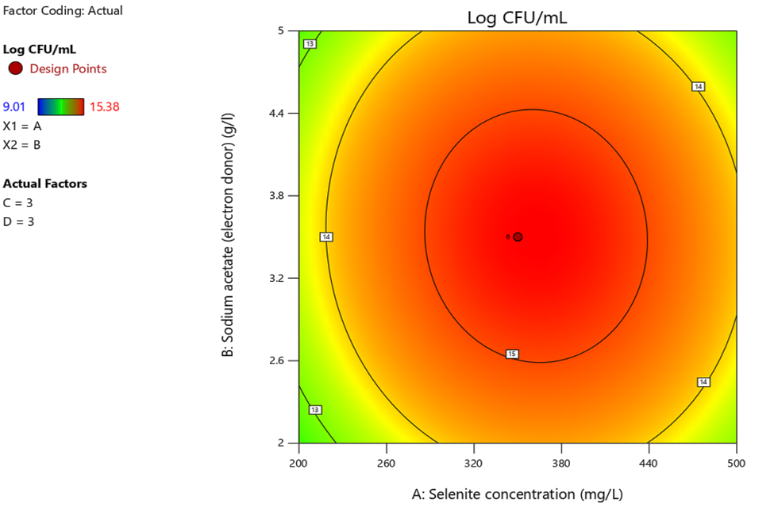** | **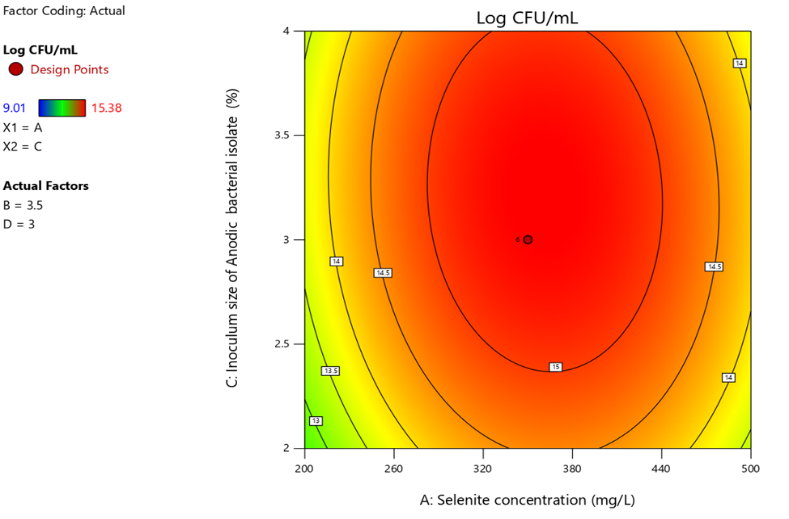** | **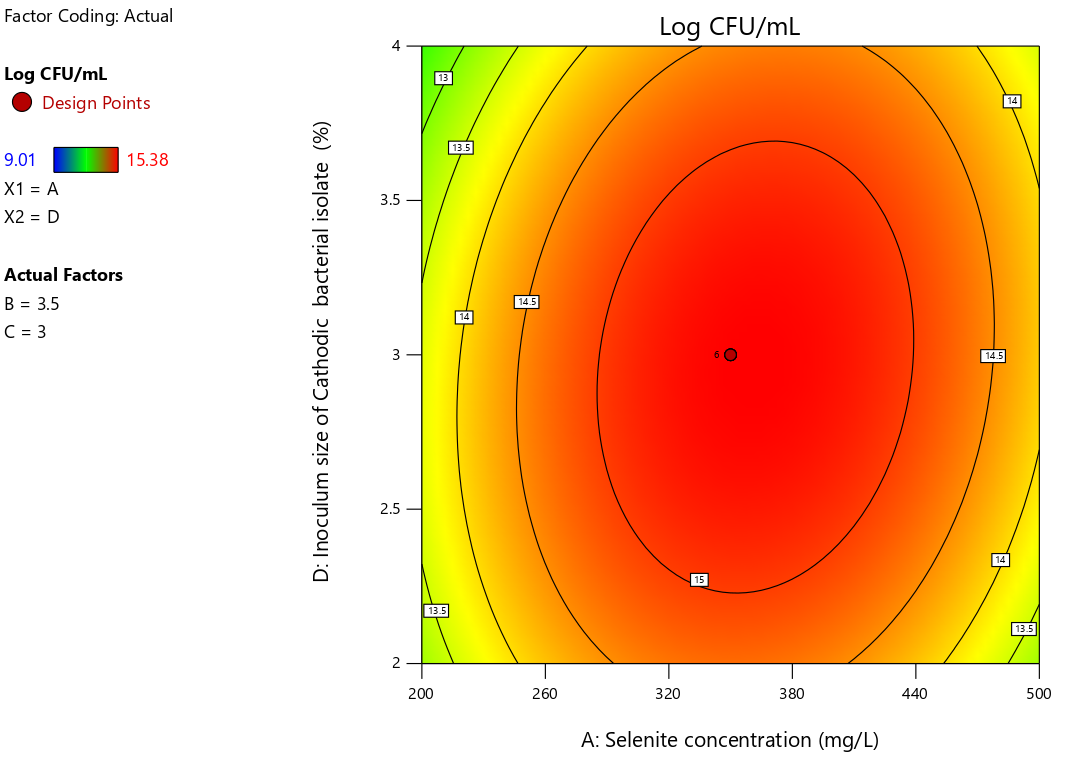** |  |
| **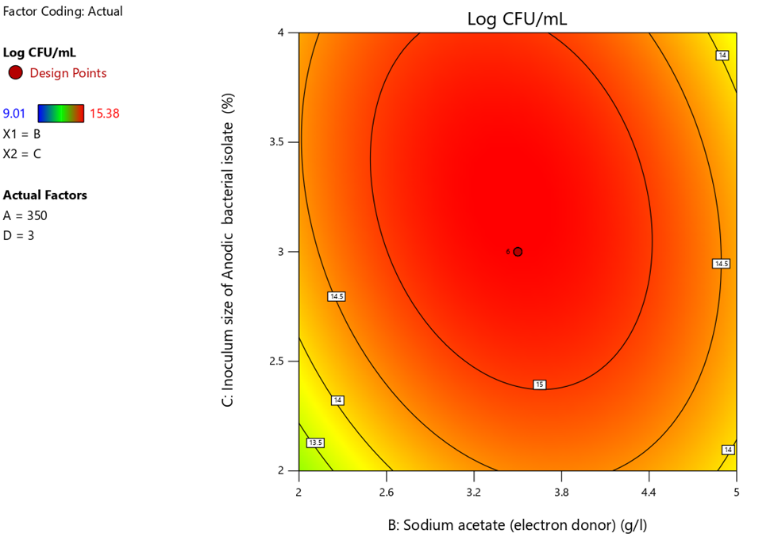** | **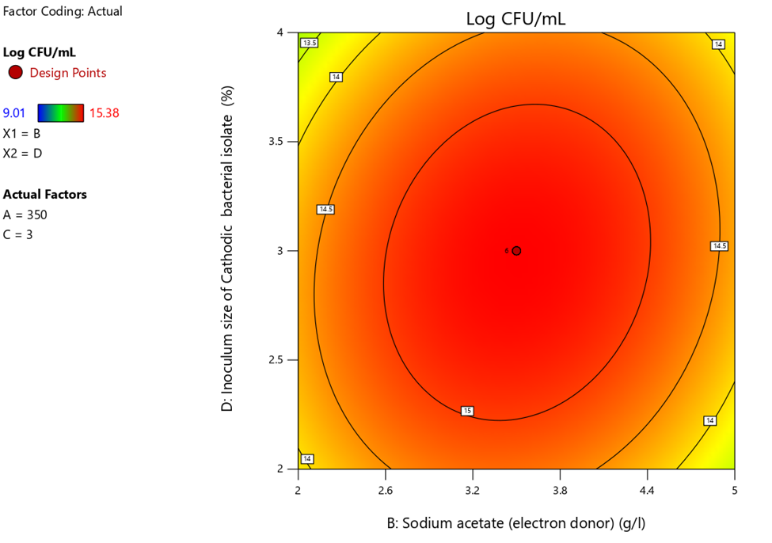** |  |  |
| **(b)** |  |  |  |
| **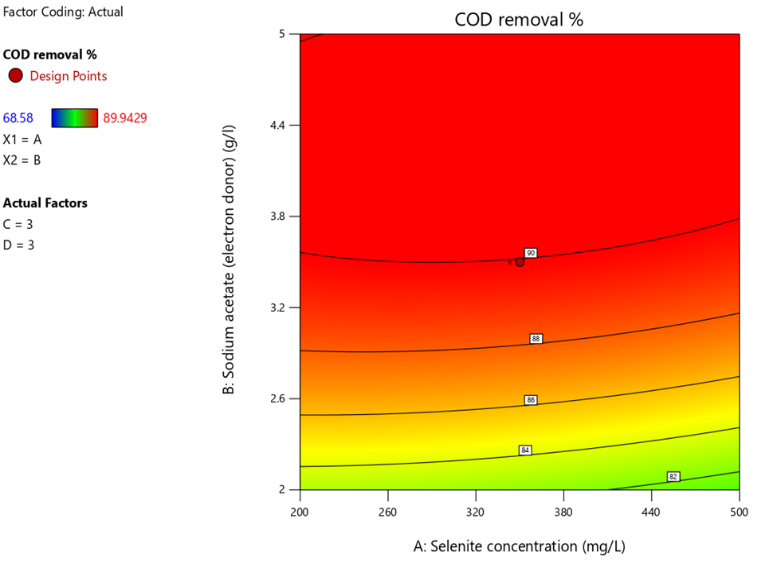** | **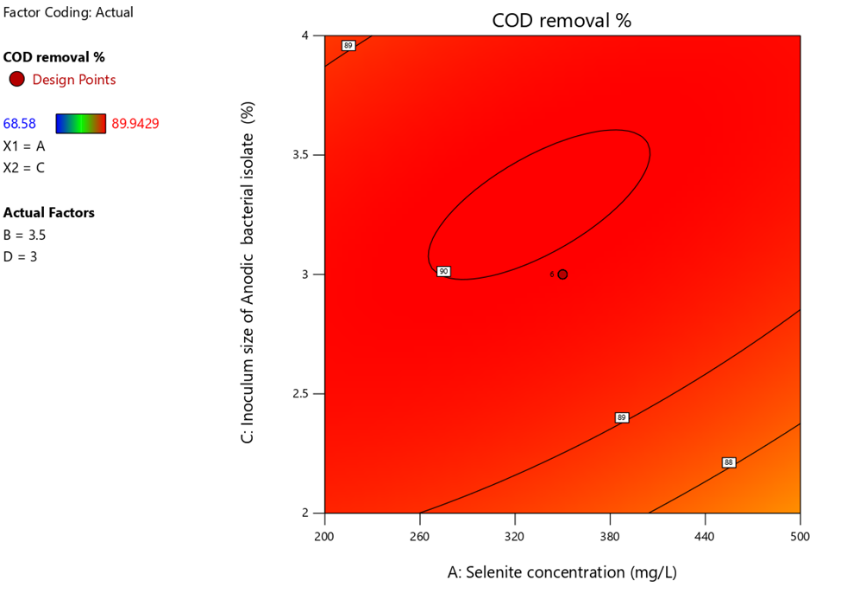** | **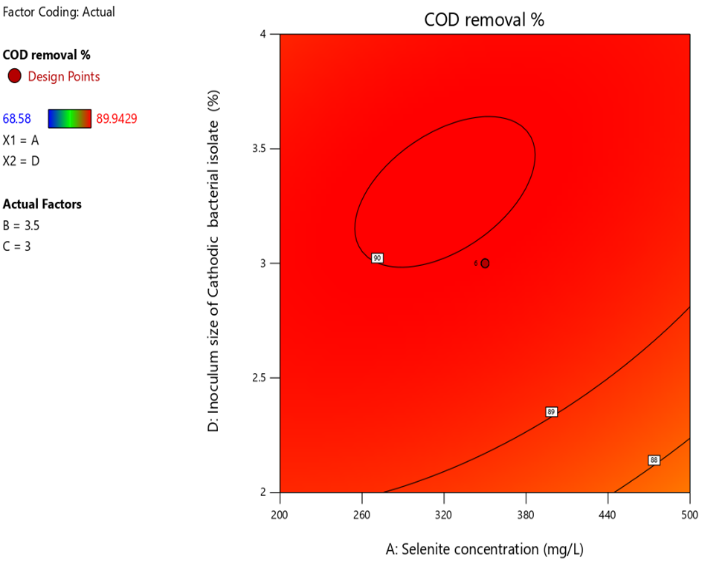** |  |
| **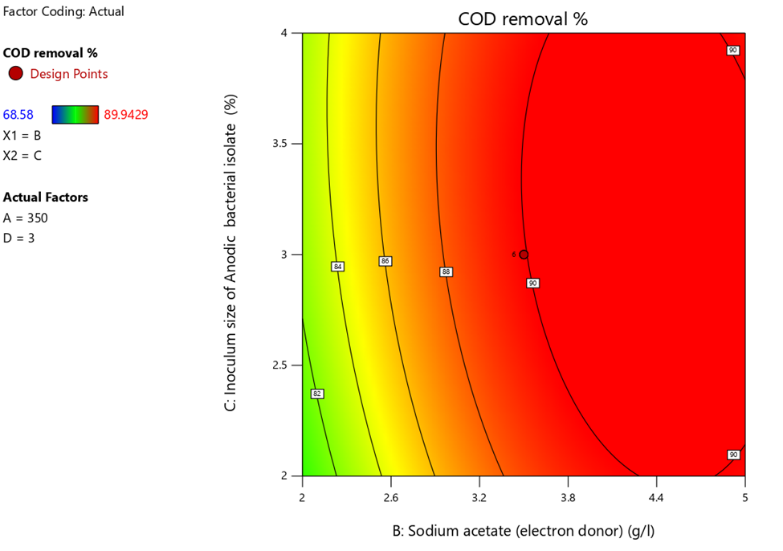** | **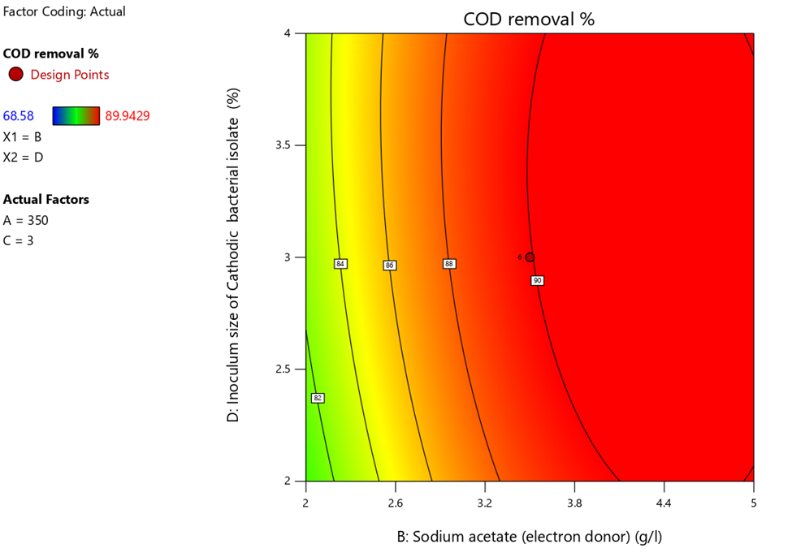** |  |  |
| **(c)** |  |  |  |
| **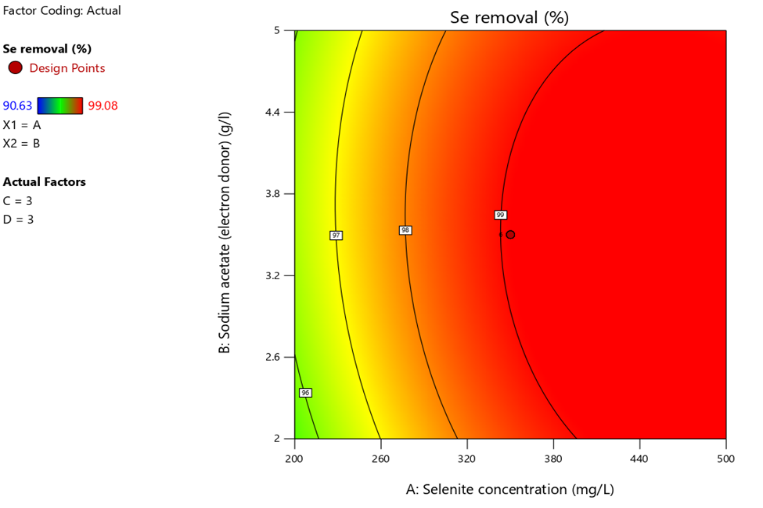** | **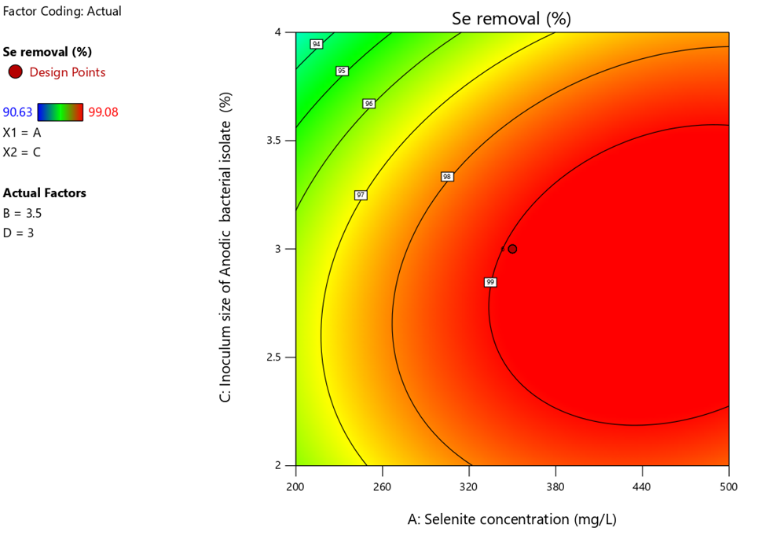** | **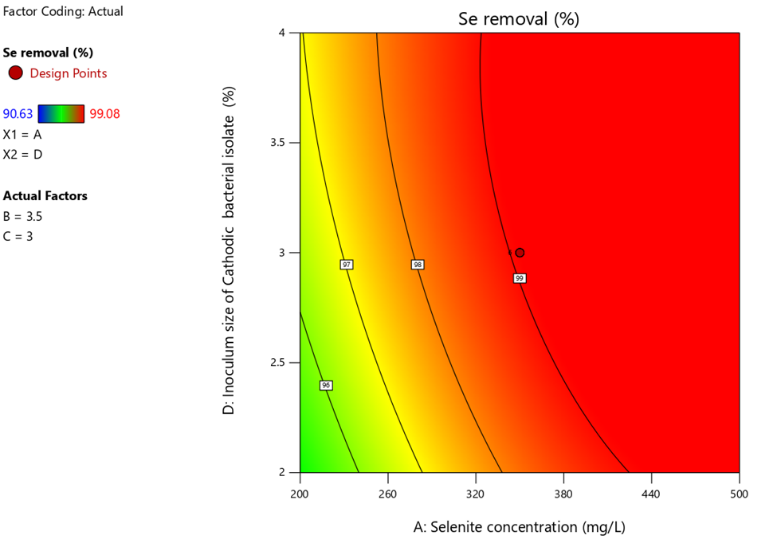** |  |
| **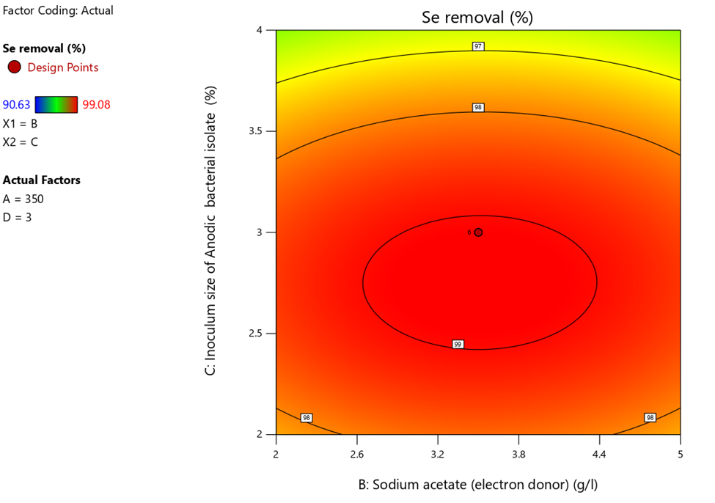** | **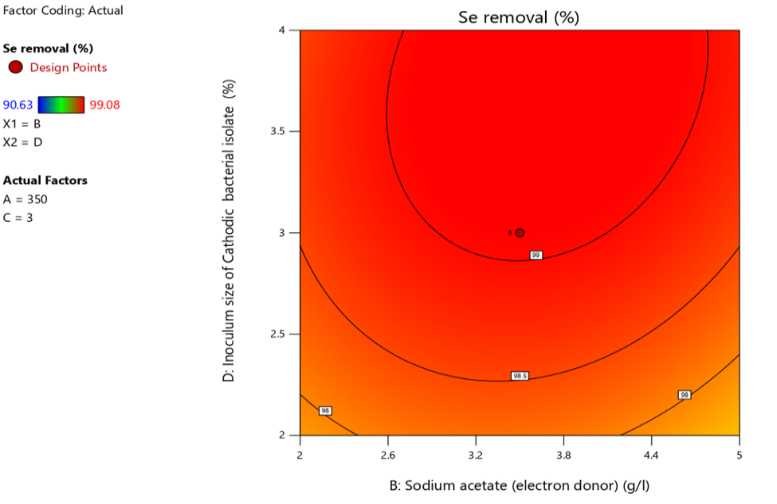** |  |  |

**Fig. S1**

2D contour plot for Log CFU mL^-1^ **(a)**, COD removal (%) **(b)**, and Se removal (%) **(c)**.
